# Efferent compared to afferent neural substrates of the vergence eye movement system evoked via fMRI

**DOI:** 10.3389/fnins.2024.1497326

**Published:** 2025-01-08

**Authors:** Ayushi Sangoi, Farzin Hajebrahimi, Suril Gohel, Mitchell Scheiman, Tara L. Alvarez

**Affiliations:** ^1^Vision and Neural Engineering Laboratory, Biomedical Engineering, New Jersey Institute of Technology, Newark, NJ, United States; ^2^Department of Health Informatics, Rutgers University School of Health Professions, Newark, NJ, United States; ^3^Pennsylvania College of Optometry at Drexel University, Philadelphia, PA, United States

**Keywords:** vergence, fMRI, sensorimotor, afferent, efferent

## Abstract

**Introduction:**

The vergence neural system was stimulated to dissect the afferent and efferent components of symmetrical vergence eye movement step responses. The hypothesis tested was whether the afferent regions of interest would differ from the efferent regions to serve as comparative data for future clinical patient population studies.

**Methods:**

Thirty binocularly normal participants participated in an oculomotor symmetrical vergence step block task within a functional MRI experiment compared to a similar sensory task where the participants did not elicit vergence eye movements.

**Results:**

For the oculomotor vergence task, functional activation was observed within the parietal eye field, supplemental eye field, frontal eye field, and cerebellar vermis, and activation in these regions was significantly diminished during the sensory task. Differences between the afferent sensory and efferent oculomotor experiments were also observed within the visual cortex.

**Discussion:**

Differences between the vergence oculomotor and sensory tasks provide a protocol to delineate the afferent and efferent portion of the vergence neural circuit. Implications with clinical populations and future therapeutic intervention studies are discussed.

## 1 Introduction

Binocular eye movement coordination is a critical skill needed for near vision, which includes reading and working on small handheld electronic devices. Vergence eye movements mediate the change within the plane of fusion either towards or away from the individual by using the disjunctive inward or outward rotation of the eyes (Scheiman and Wick, 2013). The ocular motion ensures that the target image is projected onto the fovea of each eye. Vergence eye movements are used often throughout the day. For example, vergence eye movements are mediated by a child in a classroom looking between the chalkboard and their notebook or by a baseball player tracking a fastball. The vergence eye movement neural system receives afferent information from the eyes to track objects at different spatial depths. This afferent signal traverses from the retinas, through the lateral geniculate nucleus in the thalamus to the visual cortices (Suleiman et al., 2020). It then translates the sensory afferent information into an efferent neural response for the final common system to generate a vergence eye movement response. Understanding the efferent motor component of the vergence system and how it differs from the afferent sensory component of the oculomotor vergence system in participants with normal binocular vision is critical for the basic knowledge of vergence eye movement neural substrates. The basic science of how eye movements are stimulated in humans is a necessary first step to understanding how the neural substrates of those with binocularly normal vision differ from those with vergence dysfunctions.

This study seeks to identify the neural substrates involved in the afferent and the efferent portions of vergence eye movements neural circuit using functional magnetic resonance imaging (fMRI) in young adults with normal binocular vision. Vergence movements have been studied in primates using single-cell recordings (Busettini et al., 2009; Mays et al., 1986; Mays and Gamlin, 1995; May et al., 2018; Ward et al., 2015) and functional imaging (Ward et al., 2015). In humans, the vergence oculomotor system’s neural components have been studied via case reports in patients with vergence dysfunction (Rambold et al., 2005b; Rambold et al., 2005a; Anagnostou et al., 2021; Sander et al., 2009), fMRI (Alkan et al., 2011b; Alvarez et al., 2010; Zhang et al., 2023; Morales et al., 2020b; Fogt et al., 2023), functional near-infrared spectroscopy (Yaramothu et al., 2020), magnetoencephalography (Mitsudo et al., 2022), and transcranial magnetic stimulation (Kapoula et al., 2001; Coubard and Kapoula, 2006). Together, these studies support that the vergence response is facilitated by the frontal eye fields (FEF), supplementary eye field (SEF), parietal eye fields (PEF), cerebellar oculomotor vermis (OMV), midbrain, pons, and primary visual cortex (V1).

Within prior modalities, fMRI is an effective tool for determining the differences in patient populations and neurologically normal individuals (Alvarez et al., 2021). In addition, the effectiveness of therapeutic interventions can be quantified through functional activity and connectivity in longitudinal studies (Su et al., 2018; Alvarez et al., 2020b; Alvarez et al., 2024). Though functional imaging studies have investigated the different types of oculomotor movements (Fogt et al., 2023; Alkan et al., 2011a), a current gap in the literature is delineating the vergence oculomotor system into its afferent sensory and efferent motor components. This information is critical to understanding convergence dysfunctions like convergence insufficiency, where a person cannot comfortably sustain convergence. Convergence is a critical skill for reading, which is becoming increasingly important in our society as we become more dependent on small handheld electronic devices. Convergence insufficiency is the most common binocular dysfunction in the adolescent and young adult population, present in about 5–12% of the general population (Cooper and Jamal, 2012; Porcar and Martinez-Palomera, 1997; Nunes et al., 2019; Wajuihian and Hansraj, 2016; Rouse et al., 1999; Hussaindeen et al., 2017; Davis et al., 2016; García-muñoz et al., 2016), and about half of the patients with brain injuries (Master et al., 2022; Master et al., 2016; Ciuffreda et al., 2008; Hunt et al., 2016; Alvarez et al., 2012; Scheiman et al., 2021; Gowrisankaran et al., 2021; Capó-Aponte et al., 2012; Wiecek et al., 2021), patients with psychological dysfunctions (Chrobak et al., 2020; Bolding et al., 2014), and more likely to occur in patients with attentional issues (Borsting et al., 2005; Granet et al., 2005). A better understanding of the differing etiology of these convergence issues can lead to more targeted therapeutic interventions that concentrate on specific neural substrates of the vergence system that are not at full functional capabilities and may improve with therapy.

The functional activity of the efferent vergence oculomotor neural substrates has been published, and the stimulation is reliable (Morales et al., 2020a). Yet, the vergence system has not been delineated into its separate efferent and afferent portions. This study will address this gap via scanning protocols that stimulate the afferent vergence system and the afferent with efferent systems so that the difference between these datasets will show the efferent system only. This study tests the hypothesis that when taking the difference between the activity on both scans, the afferent activation alone is subtracted from the afferent and efferent activation together, leaving just the efferent functional activity centered in the FEF, SEF, PEF, and OMV. This will be used as a foundation for understanding the etiology of convergence issues in clinical populations in the future.

## 2 Methods

### 2.1 Participants

All participants provided written informed consent for adults or written assent with legal guardian consent for minors, which was approved by the Institutional Review Boards of New Jersey Institute of Technology and Rutgers University in accordance with the Declaration of Helsinki. Participants were recruited from the New Jersey Institute of Technology in Newark, NJ, United States, which has a diverse population. The study included 31 adolescents to young adult binocularly normal participants between the ages of 15 and 22 years of age. One participant did not finish the protocol. Participants had a tutorial session prior to the experiment to allow them to practice the visual protocol.

### 2.2 Sensorimotor optometric exam

Each participant had a sensory-motor optometric exam from an optometrist (co-author MS) to ensure normal binocular vision. The following optometric tests were administered: best corrected visual acuity both at distance and at near; refraction; convergence insufficiency symptoms survey (CISS) (Rouse et al., 2004; Rouse et al., 2009); negative and positive fusional vergence (NFV and PFV) at near (step vergence procedure); local and global stereopsis (Randot Stereotest); near point of convergence; the cover test with prism neutralization at distance (6 meters) and near (40 centimeters); vergence facility at near (12∆ base-out/3∆ base-in), the monocular amplitude of accommodation, and monocular accommodative facility (+2/-2D lenses) at near.

These measures have been utilized in previous clinical trials to evaluate for potential vergence eye movement dysfunctions (Alvarez et al., 2020b; Scheiman et al., 2008; CITT-ART Investigator Group, 2019). Eligibility criteria for normal binocular vision were defined as the following; best-corrected visual acuity of 20/25 or better in both eyes, normal near point of convergence of less than 6 cm measured from the bridge of the nose, amplitude of accommodation within the normal range (based on Hofstetter’s rule) (Hofstetter, 1944), local stereopsis of less than 70 s of arc and global stereopsis of less than 500 s of arc, normal positive and negative fusional vergence based on Sheard’s criterion (fusional vergence equals at least twice the magnitude of the near phoria) (Sheard, 1930). The CISS is a 15-question Likert survey to determine the levels of convergence-related symptoms, where a higher score means the patient is more symptomatic. A CISS of less than 21 points for adults and less than 16 for children is considered asymptomatic in this population (Rouse et al., 2004). Only asymptomatic participants with normal binocular vision were enrolled in this study.

Demographic data were collected by the examiners, including age, sex, race, ethnicity, dominant hand, self-reported attentional issues, and athletic status. Participants were excluded if they had any other oculomotor or retinal dysfunctions like manifest or latent nystagmus; a brain injury or concussion; physiological or attentional dysfunctions; history of Lasik or any other eye surgery such as strabismus or refractive surgery; eye injury; or inability to complete the study tasks. Participants were also excluded if they had a large refractive error of more than 7D myopic or hyperopic or anisotropia of more than 1.5D between the eyes.

### 2.3 Materials

**Figure 1:**
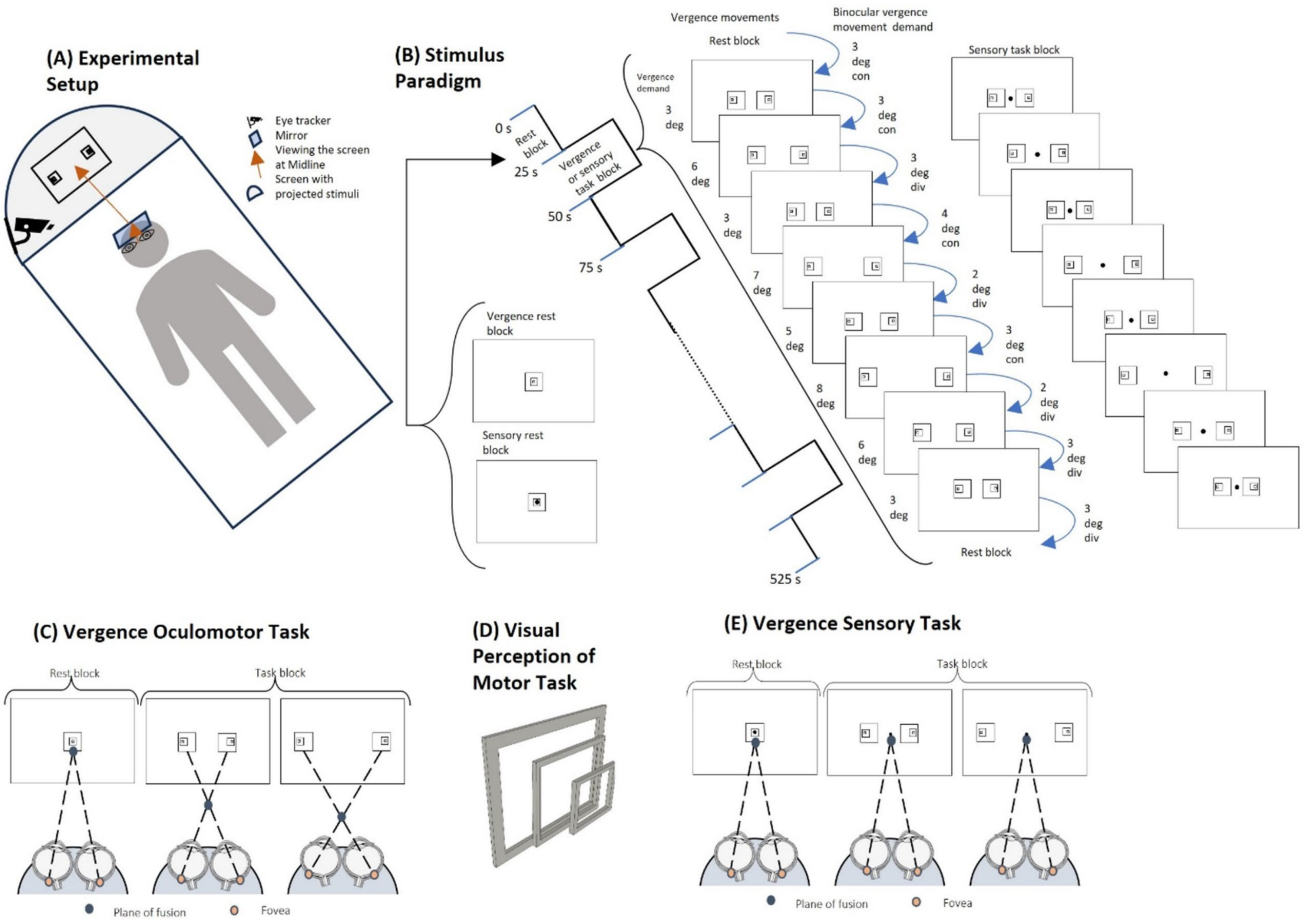
Experimental set-up and stimuli. **(A)** The experimental setup shows the participant lying on the fMRI table with the angled mirror in front of the participant’s eyes. The participant is looking up at the projector screen that is reflected onto the mirror within the bore of the MRI. The eye tracking apparatus is attached to the projector screen light to capture the right eye position. **(B)** The stimulus paradigm uses a 25-s interleaved block design for both the vergence motor and sensory task sequences. The rest blocks have one set of concentric boxes. The vergence oculomotor task consists of two sets of eccentric boxes where each task block consists of 9 vergence eye movements. The sensory task block looks similar but does not have any vergence demand as the participant is asked to visually fixate on the dot in the center of the projection for the duration of the experiment. **(C)** For the vergence oculomotor task, the participant performs free fusion vergence motions whenever there are two sets of eccentric boxes on the screen in order to make the outer boxes overlap. A greater horizontal distance stimulates more vergence disparity between the sets of boxes and hence stimulates a larger prismatic demand to mediate a larger vergence eye movement to fuse the targets. **(D)** As the squares are eccentric, not concentric, the visual perception of overlapping the sets of squares gives a 3D effect, so the participant has visual feedback they are correctly performing the task when the inner box projects out towards the participant more than the outer box. **(E)** The visual stimuli of the sensory task look the same as the oculomotor task, but the participant is asked to continue fixating on the dot in the center, eliminating the vergence oculomotor movements.

In this study, fMRI data were acquired with a 3 T Siemens PRISMA (Siemens Medical Solutions, Parkway Malvern, PA, United States) at Rutgers University Brain Imaging Center (RUBIC) (Newark, NJ). All participants were scanned inside a 64-channel head and neck coil with an EyeLink-1,000 compatible mirror mounted to it. Monocular right eye tracking was recorded with the EyeLink-1,000 infrared within-MRI-bore eye tracking system (SR Research, Kanata, ON, Canada) with a sampling rate of 250 Hz and a spatial resolution of 0.25 degrees to ensure each participant was performing the task (Figure 1A). RUBIC is a shared resource imaging center, hence only monocular eye tracking is permitted so that the eye tracker does not obstruct the view for other research experiments. During training, a binocular ISCAN eye tracker (Wolbum, Mass, USA) outside the imaging center was used to objectively record the left and right eye positions at 240 Hz with a spatial resolution of 0.1 degrees.

Within the magnet bore, the participant was aligned so that their midline was centered symmetrically within the head coil. Participant centering was important to maintain consistent and clear eye movement responses, track data acquisition via an infrared emitter and detector, and present visual stimuli symmetrically. Participants viewed a mirror placed directly above them, angled in front of their eyes, to see the projector screen at an optical distance of 95 cm away. This distance was measured from the nasion of the participant up to the mirror, measured at 15 cm, and 80 cm from the mirror to the center of the projector screen.

The projected image of the within-bore screen measured 32 cm by 18 cm. The visual stimuli were programmed using MATLAB (2020) and Psychtoolbox (Kleiner et al., 2007; Brainard, 1997), with the addition of the EyeLink Toolbox from SR Research (Cornelissen et al., 2002). The stimuli were projected from a computer within the MRI control room, with the computer screen resolution measuring 1,920 by 1,080 pixels.

### 2.4 Eye movement acquisition during imaging

EyeLink uses a nine-point calibration system to convert eye position in pixels into angular position. Calibration was performed for each subject before collecting any eye movement data for the entire task-based and anatomical scanning protocol. Within the MRI-compatible EyeLink system, the video image of the eye with the real-time eye movement position signal was observed by the operator. The operator was in communication with the subject via an intercom. If a participant blinked or did not fixate on the calibration target, then that calibration point was acquired again. The operator gave verbal feedback to the subject if the subject was having difficulty with the calibration sequence.

### 2.5 Vergence oculomotor stimuli

The visual stimuli for this study have been used in prior clinical trials (Alvarez et al., 2020a). The visual stimuli utilized a block design consisting of interleaved periods of sustained visual fixation and periods of vergence oculomotor demands, shown in Figure 1B. The rest block consisted of sustained visual fixation for 25 s. Each participant viewed a single set of concentric squares for the rest block (Figure 1C), where the fusion plane is positioned at the screen’s distance of 95 cm away. For the task block, two sets of eccentric squares placed at different horizontal distances from the center created different vergence oculomotor demands (Figure 1C). Upon successfully fusing the targets for the given vergence demand, the inner boxes when overlapped will protrude closer to the person as compared to the outer boxes, creating a 3D visual feedback for the participants. The off-center inner boxes provoke more vergence disparity compared to the outer boxes within the free-fusion stereogram. Hence, participants observe the perception of depth which is visually displayed in Figure 1D. For the task block, there was a set of nine vergence oculomotor demands to stimulate vergence eye movements for the participant, (Figure 1B). The different vergence demands followed a pseudo-random sequence of binocular vergence demands, ranging from 2 degrees to 4 degrees in either the convergent (inward eye rotation) or divergent (outward eye rotation) directions. In addition to varying the vergence demands of the stimulus presentations, the time when the vergence demand shifted varied between 2.5 and 3.5 s. Anticipatory cues are reduced by varying the magnitude of the stimulus and the time when the stimulus is presented. Anticipatory cues have been reported to stimulate predictive neural substrates (Alvarez et al., 2010). During each task block, symmetrical binocular disparity vergence step stimuli were presented with the binocular vergence demands in the following order: 3-degree convergent (CON), 3-degree CON, 3-degree divergent (DIV), 4-degree CON, 2-degree DIV, 3-degree CON, 2-degree DIV, 3-degree DIV, and 3-degree DIV. The sequence of a pair of eccentric boxes was presented for 25 s for each task block, and each task block was repeated 10 times, interleaved with 11 rest blocks (Figure 1B).

Keeping the vergence demand at a far disparity reduced the effect of confounding variables, such as phoria adaptation on the vergence neural activity (Kim et al., 2011) and increased the magnitude of neural activation (Alvarez et al., 2010). Participant compliance with the experimental task was improved by having the participants be presented with the free fusion visual stimuli before the imaging experiment for a few minutes in the laboratory to learn how to fuse the eccentric squares by co-author AS. All participants successfully fused the visual stimuli during training and practiced this technique before being imaged, and hence were eligible for the study. This was verified through verbal feedback of the 3-dimensional perception of the stimuli in Figure 1D, and confirmed with eye tracking during the imaging scan. Although there are other methods of eliciting vergence eye movements, (Chang et al., 2020) this form of free fusion stereograms has been used in the clinical management of binocular vision dysfunctions by optometrists (Scheiman and Wick, 2019). Repeated practice enables the participants to learn how to free-fuse the targets to ensure participant compliance with the experimental task (Ravisankar et al., 2024).

**Figure 2:**
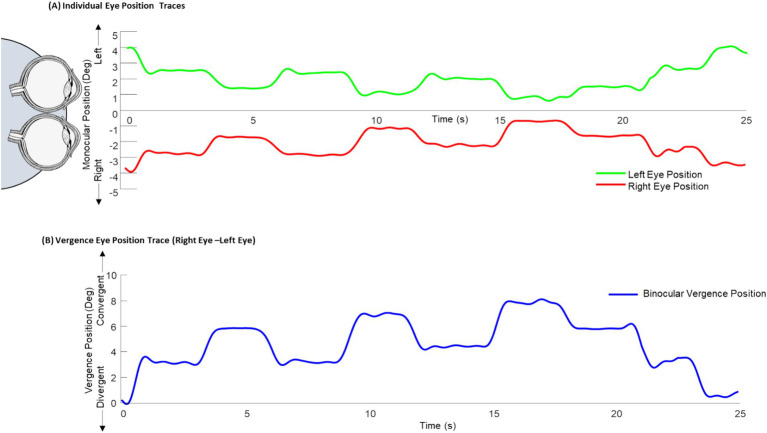
Binocular eye tracking during free-fusion task. **(A)** Binocular eye tracking was done prior to the imaging experiment where the monocular left eye (green) and right eye (red) position traces were collected over one free fusion epoch. **(B)** The difference between the right and left eye position traces were calculated for the binocular vergence position trace (blue).

Binocular eye tracking was acquired before the imaging experiment to ensure the participants could mediate vergence eye movements. Objective vergence responses were collected using an ISCAN infrared eye movement monitor with four monocular calibrations per eye. All participants were able to converge and diverge their eyes which was confirmed via a binocular objective eye movement tracker before the imaging experiment. Figure 2 displays one epoch of the eye movements during the training of the free fusion task with binocular eye tracking. Eye position was recorded with a 240 Hz ISCAN video-based infrared binocular eye-tracker with a resolution of 0.1 degrees. Calibration was performed using four monocular positions for each eye. The monocular left eye position (green trace Figure 2A), the monocular right eye position (red trace Figure 2A), and the difference of these monocular eye positions or binocular vergence response (blue trace Figure 2B) show the binocular vergence position reaches the stimulated binocular demand depicted in Figure 1B.

### 2.6 Vergence sensory stimuli

The stimuli for the vergence sensory experiment matched the vergence oculomotor stimuli with the addition of a dot in the center of the display, Figure 1E. The participant was instructed to visually fixate on the dot throughout the experiment. Fixation on the central dot does not change their task for the rest block, but for the task block, central fixation ensures that they maintain that sustained fixation at the plane of fusion of the screen, as opposed to making the vergence movements. Other than the addition of a small dot to allow the participants a visual point to fixate, no other variable was altered between the vergence oculomotor stimuli and the vergence sensory task, including time, color, and sequence. This ensures consistency between the two sets of stimuli so that the afferent and efferent stimulation datasets can be compared.

### 2.7 Scanning parameters

All scans were done in a one-hour imaging session, where the participant was not able to leave during the scan. After the calibrations, the participant went through the vergence oculomotor protocol, immediately followed by the vergence sensory protocol, in which the operator communicated with the participant about the upcoming task, to ensure the participant was aware of what they would be asked to do.

A Magnetization Prepared—RApid Gradient Echo (MP-RAGE) was acquired as a high-resolution anatomical reference volume (repetition time (TR): 1,900 ms, echo time (TE): 2.52 ms, T1: 900 ms, flip angle: 9 degrees, field of view (FOV): 256 mm, and spatial resolution: 1.0 mm x 1.0 mm x 1.0 mm x 1.0 mm). This was acquired with 176 slices.

The functional imaging was captured with a multiband echo planar imaging sequence (TR: 720 ms, TE: 33 ms, FOV: 192 mm, flip angle: 90 degrees, spatial resolution: 3.0 mm x 3.0 mm x 3.0 mm) with 56 slices and 730 volumes. This applied to both the vergence oculomotor stimuli and the vergence sensory stimuli.

### 2.8 Pre-processing pipeline

Preprocessing of the blood oxygenation level-dependent (BOLD) datasets followed the pipeline delineated in prior studies (Morales et al., 2020b; Alvarez et al., 2021; Morales et al., 2020a; Alvarez et al., 2020a; Alvarez et al., 2019; Hajebrahimi et al., 2023). Upon retrieval of the data from the imaging center’s server, the NIFTI images were extracted, and the data were sorted into the BIDS format (Gorgolewski et al., 2016).

Pre-processing was completed using SPM 12 (Wellcome Centre for Human Neuroimaging, UCL, London, United Kingdom) under default parameters unless otherwise stated. During preprocessing, the datasets were realigned to the first volume to extract the motion of the participants during the scan. The realigned volumes were co-registered to their anatomical MP-RAGE files. Motion artifact analysis was performed for each 4-dimensional file. After calculating the framewise displacement of each set of volumes, if the mean motion exceeded 0.5 mm or more than 20% of the time points had greater than 2 mm of motion, then that dataset was excluded from all other analyses (Leonard et al., 2017).

Segmentation was performed with anatomical files to extract the white matter (WM) and cerebrospinal fluid (CSF), and tissue probability maps at the threshold of 0.97 were used to extract masks for each tissue type. This also created a deformation field file that is used during normalization. Principal component analysis was used to extract the first five components from both the CSF mask and the WM mask. Normalization was done to transform each participant’s anatomical and fMRI datasets using the previously mentioned deformation field file into standardized Montreal Neurological Institute (MNI) space using the MNI152 template (Fonov et al., 2011). Resampling to 2 mm x 2 mm x 2 mm voxels was done with a 4th-degree b-spline interpolation function.

Regression of 34 nuisance variables was done to minimize noise from head motion and physiological signals. This includes the six-movement coefficients (x, y, z, yaw, pitch, roll), with their six quadratics as well as six auto-regressors and their six quadratics for a total of 24 motion-related variables. Additionally, the five principal components of each CSF and WM total 10 physiological signal-related nuisance variables (Friston et al., 1996; Yan et al., 2013). After the regression of all 34 of those variables was completed, a high pass filter was applied with a cutoff frequency of 0.01 Hz. Smoothing was done with a 6-mm full width at half maximum kernel.

### 2.9 Whole-brain functional maps and group-level statistics

Whole brain voxel-wise functional activation maps were generated from a general linear model. In SPM, the default first-level analysis utilizes the canonical hemodynamic response function (HRF) waveform, a double gamma function, to model the beta weight for each voxel of task block compared to the rest block within each fMRI data. One-sample *T*-tests were performed for each of the stimulus types, vergence oculomotor and vergence sensory, to get group-level activation maps. Additionally, a paired *T*-test was performed for each participant’s vergence oculomotor and vergence sensory activation maps. These maps were thresholded at *p* < 0.05 with family-wise error (FWE) correction for multiple comparisons (Han and Glenn, 2018) and overlaid on the MNI anatomical images for ease of viewing the data. Correlation analysis using Pearson’s correlation coefficient was performed on the beta weights for the regions with significant differences between the vergence oculomotor and vergence sensory datasets and the optometric clinical parameters.

## 3 Results

### 3.1 Clinical optometric exam

Among the participants screened for the study, 30 participants were recruited, and all had normal binocular vision. All participants agreed and enrolled in the study. Correcting for motion artifact required the removal of two participants whose movement exceeded the prior established motion artifact criteria for either their vergence oculomotor or vergence sensory scans. For the vergence oculomotor scan, the mean motion across all participants who remained after the motion artifact check was 0.047 ± 0.015 mm, and for the vergence sensory scan, the mean motion was 0.047 ± 0.017 mm. A paired *T*-test of each participant’s average movement in oculomotor vergence and vergence sensory tasks showed no significant difference [T (27) =0.07; *p* = 0.94].

Table 1 depicts the clinical optometric examination results, including all the participant’s means, standard deviations, ranges of minimum and maximum, and 95% confidence intervals for the 28 participants whose datasets were utilized to create the group-level imaging analyses.

**Table 1:** Demographics and clinical metrics of study participants.

Binocularly normal controls group *n* = 28					
	M ± SD	Min	Max	95% confidence interval	
Sex	14 ♀ 14 ♂				
Age (Years)	18.8 ± 2.0	15.0	22.0	18.0	19.5
Positive fusional vergence (∆)	27.3 ± 10.3	12.0	50.0	23.5	31.1
Negative fusional vergence (∆)	14.6 ± 3.2	10.0	25.0	13.4	15.8
Near point of convergence (cm)	3.1 ± 1.2	2.0	5.5	2.6	3.5
Visual acuity (OD)	20.2 ± 0.9	20.0	25.0	19.8	20.5
Visual acuity (OS)	20.5 ± 1.6	20.0	25.0	20.0	21.1
CISS (points)	10.8 ± 4.6	2.0	17.0	9.0	12.5
Local stereopsis (seconds of arc)	29.4 ± 12.1	10.0	59.0	24.9	33.9
Global stereopsis (seconds of arc)	267.9 ± 65.6	250.0	500.0	243.6	292.1
Vergence facility at near (cycles / min)	17.0 ± 4.5	5.0	23.0	15.2	18.9
OD spherical equivalent (D)	−0.6 ± 1.8	−6.6	1	−1.2	0.0
OS spherical equivalent (D)	−0.60 ± 1.6	−6.3	0.8	−1.2	0.0
Participant type	18 Emmetropes: 9 Myopes; 1 Hyperope; 0 Anisometrope						

Clinical and demographic table with the mean + the standard deviation for each of the clinical and demographic measures, with the minimum and maximum values as well as the 95% confidence intervals. Sex and participant types are denoted with categorical counts. ♀ – Female; ♂ – Male, OD – Right Eye, OS – Left Eye, for mean (M) and standard deviation (SD), minimum value (Min), maximum value (Max) and 95% Confidence interval.

### 3.2 Group-level functional activation maps for vergence oculomotor stimuli

**Figure 3:**
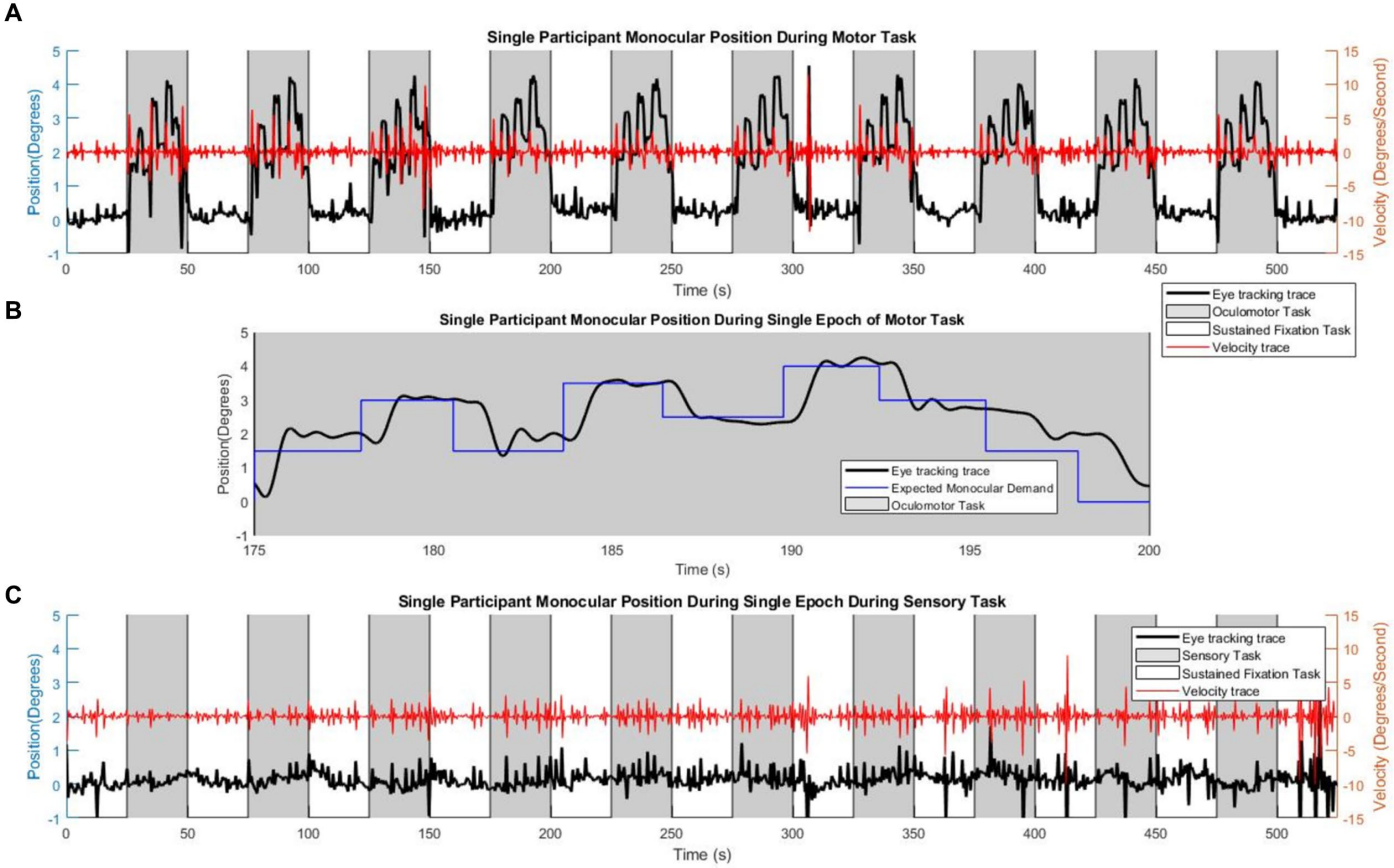
Single participant eye movement traces. Single participant eye movement traces. **(A)** An example of a single participant performing the motor task within the scanner, where the gray shading signifies the time of the oculomotor ‘task’ block, and the white shading represents the time of the sustained visual fixation portion of the ‘rest’ block sequence. The red trace is the velocity corresponding to the eye-tracking trace. **(B)** An eye movement epoch within the oculomotor task, with the visual stimulus (blue trace) signifying the number of degrees of monocular demand. **(C)** The sensory task, where the participant maintains visual fixation upon the center dot. The red trace is the velocity corresponding to the eye-tracking trace.

Individual adherence to the tasks was confirmed with visual inspection of the eye movement traces. All 28 participants mediated vergence responses during the vergence oculomotor task and kept steady eye position fixation during the vergence sensory task. An example of a single subject eye movement trace is shown in Figure 3. This subject completed all of the oculomotor tasks during the task blocks, as shown by the black trace with the axis on the left side of the figure in Figure 3A. This is confirmed by the velocity trace in red with the axis on the right side of the figure. To reveal the demand of the task as compared to the eye movements, Figure 3B has the stimulated monocular demand in blue, within the magnified epoch of one task block of eye movements. For comparison, the eye movement trace of the sensory task (black trace with left axis) is shown in Figure 3C, with its’ corresponding velocity trace (red trace with right axis).

**Figure 4:**
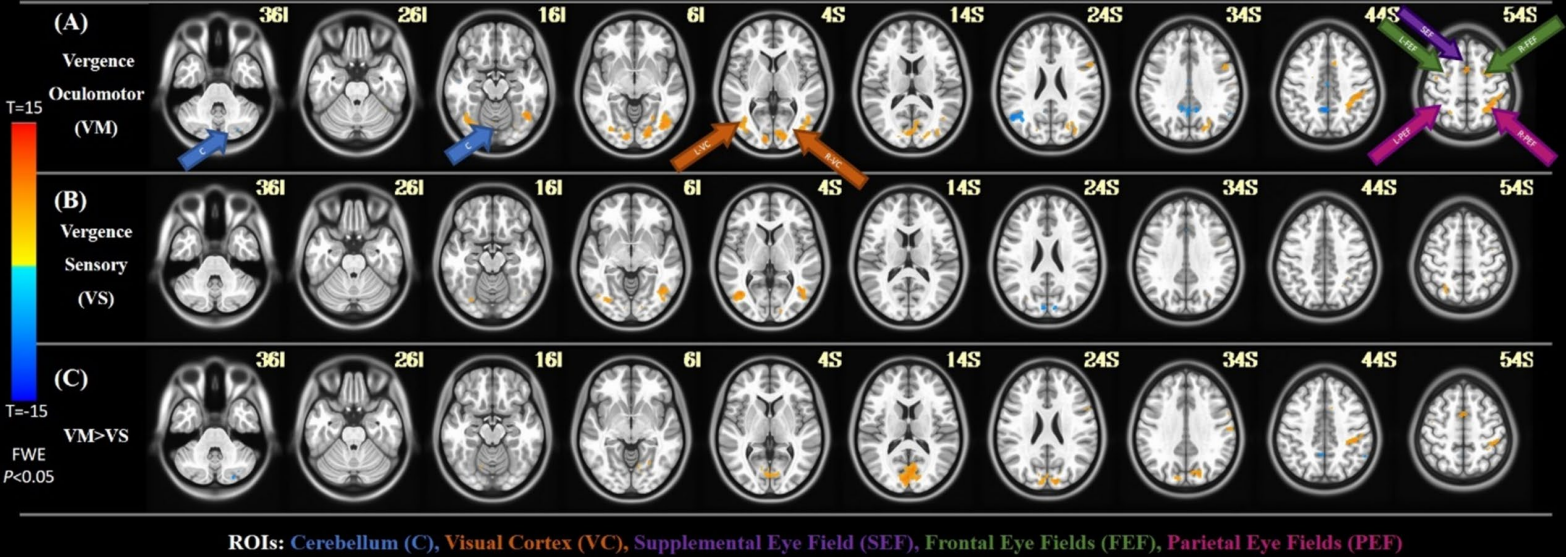
FMRI results. **(A)** The activation of the group-level average for the vergence oculomotor task. **(B)** The activation of the group-level average for the sensory test. **(C)** The group-level activation with a paired t-test comparison between the vergence oculomotor task as well as the sensory task.

Analysis of Functional NeuroImages (AFNI) is used for the visualization of the brain activity in the group-level activity maps with a threshold of FWE *p* < 0.05 (Cox, 1996). Figure 4 shows the results with the key ROIs labeled, including the cerebellum (C), Visual Cortex (VC), Supplemental Eye Field (SEF), Frontal Eye Fields (FEF), and Parietal Eye Fields (PEF). In the first row, the activation from the vergence oculomotor task is shown, with greater than 10 voxel clusters of positive activation in the VC, SEF, FEF, and PEF. There are also greater than 10 voxel clusters of delayed or negative activation in the cerebellum and precuneus. In the second row, one-sample T-tests of the vergence sensory activation are shown, with greater than 10 voxel clusters of positive activation in the VC and PEF. Greater than 10 voxel clusters of delayed or negative activation are found in the cuneus. The third row shows the paired T-test activation where the vergence oculomotor exceeds the vergence sensory activation, with a correction for multiple comparisons FWE set at a threshold of *p* < 0.05. Table 2 shows the peak MNI coordinates of all positive regions where VM activation was greater than VS activation that survived the paired T-test correction for multiple comparisons with FWE *p* < 0.05 using the T threshold from SPM and a cluster size of greater than 10 voxels within AFNI using the T threshold from SPM.

**Table 2:** Vergence oculomotor (VM) vs. vergence sensory (VS) paired *T*-Test, results corrected for multiple comparisons with family wise error (FWE, *p* < 0.05, cluster size >10).

Contrast	Number of voxels	Peak MNI coordinate X Y Z			Brain regions
VM > VS	1,130	−2	−82	12	Primary Visual Cortex, Second Visual Area, Left Calcarine Gyrus, R Cuneus, R V3A
VM > VS	251	44	−36	58	Right Postcentral Gyrus, Right Middle Temporal Gyrus, R Parietal Eye Field
VM > VS	66	−16	−78	32	Left cuneus
VM > VS	58	0	6	54	L SMA, Supplementary Eye Field
VM > VS	52	62	−16	30	R Parietal area F part t, R Postcentral Gyrus
VM > VS	32	60	6	32	R Precentral Gyrus, R Ventral Area 6
VM > VS	29	14	−72	−10	R Third Visual Area, R Lingual Gyrus
VM > VS	29	24	−62	−8	R Ventromedial Visual Area
VM > VS	20	−22	−54	58	L Lateral Area 7A, L Superior Parietal Lobule
VM > VS	12	32	−44	52	R Inferior Parietal Lobule

The tabular results of the paired *t*-tests for the VM and VS datasets corrected for multiple comparisons with FWE *p* < 0.05, with the clusters with at least 10 voxels listed in the table. Relevant brain regions were found using the AFNI where am I function.

### 3.3 Correlation between functional activation and clinical measures

A Pearson correlation analysis was performed between the beta weights of the following regions during both the vergence oculomotor and vergence sensory tasks: left FEF, right FEF, left PEF, right PEF, SEF, and cerebellar vermis and the following clinical measures: near point of convergence, PFV, and NFV. No correlations between the functional activity and clinical measures reached the threshold of statistical significance where the r values for all analyses were *r* < 0.37 with a significance of *p* > 0.05.

## 4 Discussion

### 4.1 Comparison between vergence oculomotor and vergence sensory task-induced activation

This study is the first to dissect the neural substrates of the vergence eye movement system into its motor and sensory systems through functional neuroimaging in humans. The main difference between the vergence oculomotor and vergence sensory tasks was located within the occipital lobe in the visual cortex specifically the primary visual cortex (V1). VI is highly related to foveal visual stimulation (Baker et al., 2018). Foveal stimulation was present in the vergence oculomotor task, as the participant must continuously rotate their eyes to keep the target within the fovea of their eyes. Conversely, foveal stimulation was held constant during the vergence sensory task because the participant fixed on a single target throughout the experiment. In both humans and nonhuman primates, V3 and V3A are known to activate from the depth processing (Anzai et al., 2011; Backus et al., 2001; Adams and Zeki, 2001; Zhu et al., 2024; Rosenberg et al., 2023), which is incorporated when the participant is shifting their fixation to differing depths due to vergence eye movements during the vergence oculomotor task. Depth did not change during the vergence sensory task. V5 is implicated in motion adaptation and position interaction (McGraw et al., 2004), especially smooth pursuit versional eye movements (Coiner et al., 2019), making it key for attaining the target goals during the vergence oculomotor task. Additionally, V5, along with V1, is implicated in incoherent motion (McKeefry et al., 1997), where objects are not moving in the same direction. When the eyes cross during vergence movements, the stimuli from each of the eyes move in opposing directions, creating a form of incoherent motion. Significantly more V1, V3A, and V5 activation was observed during the vergence oculomotor experimental datasets compared to the vergence sensory experimental datasets, making them more important in peripheral, motion, and depth processing.

For this current study, significantly more negative cerebellar and precuneus BOLD activation was observed during the vergence experimental motor datasets compared to the vergence sensory datasets, making their temporal delay in the vergence oculomotor task significant. Negative activation can be interpreted as activation with a long delay. The sustained fixation experiment did not significantly stimulate BOLD functional activity within the cerebellum or precuneus. Hence, the vergence oculomotor task had more BOLD activation albeit delayed compared to the sensory experiment.

While the clinical optometric results did not significantly correlate with the beta weights from the functional imaging ROIs, we hypothesize that the reason we did not observe significance in the correlation is that the study was composed of healthy participants where the range of clinical values and functional activation was limited. We suspect if patients were included in future analyses, we may observe significant correlations between the clinical measurements and the beta weights.

### 4.2 Comparison to other vergence functional MRI and cellular recording studies

Prior research from our laboratory and another show that vergence eye movements stimulate activation in the following regions of interest: FEF, SEF, PEF, and cerebellum. Our group has used the same stimulus types previously and validated that the functional activity for the vergence oculomotor task is reliable (Morales et al., 2020a). The areas of activation for the vergence eye movements during the oculomotor task are similar to prior studies. The current study has a greater magnitude of vergence network activation than other groups (Fogt et al., 2023), which is hypothesized to be due to an increase in the number of cycles within the rest and task design (Alvarez et al., 2010). Other studies have reduced activation compared to this study which may in part be due to the predictability of the paradigm (Fogt et al., 2023) which has been shown to lead to lower activation within the vergence network (Alvarez et al., 2010). A limitation of the current study is that it had little midbrain activation due to a low signal-to-noise ratio within the deeper brain structures, making it difficult to compare to other studies with brain stem structure activation.

Primate studies support that the vergence oculomotor signal is present within the FEF (Gamlin and Yoon, 2000) and is in a similar location to the results presented here. Cellular studies also report that Purkinje cells within the cerebellar dorsal vermis mediate convergent eye movements, where paralysis of the sites in which those cells were recorded resulted in a lower peak velocity of convergence, not a complete halting of the convergent movement (Nitta et al., 2008). The cerebellum was implicated in the sensory portion of the vergence movements, which supports a lower ability to complete those movements as opposed to the inability to perform vergence oculomotor tasks. FMRI in primates confirms that there are separate areas within the same tract dedicated to different eye movements (Ward et al., 2015).

### 4.3 Comparison to other oculomotor tasks

As there is still discourse between connection and separation of the saccadic, conjunctive eye movements, and vergence, disjunctive eye movements, many of the higher level cortical functional activity is in similar locations for saccade and vergence eye movements functional activity. More literature has been published investigating the saccadic system compared to the vergence system. For the saccadic system, the SEF is involved with the planning of eye movements and facilitates the fine-tuning of the control of eye movements (Abzug and Sommer, 2017). Similarly, the SEF is active in the efferent system of this experiment as it is necessary to fine-tune those vergence eye movements that are only done during the vergence oculomotor task.

The FEFs have been studied in primates and are involved in planning and executing saccadic eye movements (Bedini et al., 2023) and vergence eye movements (Gamlin and Yoon, 2000). The present study adds further confirmation that FEF is part of the efferent portion of the vergence system because it was not significantly active in the isolated vergence sensory task but is activated in the oculomotor task experiment. In general, the FEF is proposed to generate the neural command to stimulate eye movements, as well as participate in visual attention (Vernet et al., 2014). A greater need for visual attention is present in the vergence oculomotor task, which evokes the vergence eye movements, as compared to the vergence sensory task.

The parietal eye fields (PEF) have been less studied as compared to the frontal eye fields, but a case study (Phamnguyen et al., 2022) has confirmed stimulation of the PEF is involved in horizontal conjugate contralateral eye movements. It is also key for sensorimotor integration (Kropf et al., 2019), such as incorporating motor effects into sensory inputs (Lee et al., 2013). More activation of PEF was observed in the oculomotor task compared to the sensory task in the present study which may be due to sensorimotor integration. Within that area, the medial temporal gyrus is also implicated in 2D motion and stereoscopic cue responses within non-human primates with about 10% of medial temporal neurons showing a significant effect of vergence when assessing 3D motion (Thompson et al., 2023). The stimuli used in this study’s paradigm do elicit free-fusion vergence to stereoscopic cues, however, they do not ramp from one set of demands to the next and do not have constant motion in the stimuli used within the current stimuli.

### 4.4 Vergence eye movements in patient populations

In intermittent exotropia, when one or both eye(s) deviates outwards intermittently, an fMRI study confirmed even with intermittent exotropia, the FEF, PEF, visual cortex, and cerebellum were activated in the vergence task. However, this study did not report activation from the SEF, but the stimulus for the vergence movements was also different compared to the one used within this study. In the intermittent exotropia study, the eyes each viewed the 3D stimuli with different color lenses (one blue and one red) over each eye (Zhang et al., 2023). As the SEF is implicated in planning and controlling eye movements, less feedback from the SEF may be needed for a multicolor vergence stimulus. The cerebellum was also implicated as having lower activation in the intermittent exotropia group compared to the binocularly normal controls (Zhang et al., 2023), which played a greater role in the sensory portion of the vergence circuit in this analysis. The intermittent exotropia group dataset showed lower activation in the FEF and PEF (Zhang et al., 2023), which may in part be due to the differences in the generation of motor commands in the intermittent exotropia group compared to the binocularly normal group. Research supports that patients with intermittent exotropia may have dysfunction with both sensing and mediating the vergence eye movements. Additionally, the cuneus had lower activation in intermittent exotropia than controls (Xia et al., 2024), which was also implicated in the motor portion of the vergence circuit. Future research includes using this study’s protocol that dissects the sensory from the motor portion of the vergence neural circuit to identify the degree to which intermittent patients are different from participants with normal binocular vision.

This present data can be used as a comparative dataset for patients with convergence insufficiency (CI), whether the CI is idiopathic or from traumatic brain injury or dysfunction. Currently, CI has been studied through fMRI while conducting eye movements and during resting state scans. In addition to baseline studies, longitudinal studies have shown the effects of office-based vergence and accommodative therapy or office-based placebo therapy on such functional activity and connectivity. A resting state functional connectivity analysis of CI patients undergoing office-based vergence and accommodative therapy showed many connectivity pairs involving the ROIs of this study improved connectivity after the active therapy but not with placebo therapy, showing those ROIs’ connections can be improved as well post-therapy (Hajebrahimi et al., 2024). Functional imaging research concentration on the sensory versus the oculomotor portions of the vergence system may distinguish between the underlying neural mechanism for similar signs and symptoms related to convergence disorders. This can be used to create better non-invasive or non-pharmacologic therapies for individuals based on their individual underlying convergence dysfunction.

Convergence function is affected in patient populations within the brain injury group, and patients with neurodegenerative diseases. For example, Joubert syndrome is characterized by midline cerebellar and midbrain underdevelopment. In a study with eight patients, brain imaging confirmed the lack of a cerebellar vermis activation where all of the participants had issues with conjugate eye movements, yet the functional imaging of vergence in this population has not yet been studied (Weiss et al., 2009). The cerebellum is implicated in vergence deficits in a study of patients with cerebellar lesions (Sander et al., 2009). Due to the importance of the cerebellar region in vergence oculomotor tasks, more studies are needed to determine the functional vergence circuitry and how vergence eye movements are affected due to hypoplasia of the cerebellum and midbrain.

Degenerative brain diseases like mild cognitive impairment, Alzheimer’s disease, and Parkinson’s disease have a high prevalence of vergence dysfunction. Within Alzheimer’s disease, the ability to perform vergence eye movement deteriorated with the progression of Alzheimer’s disease. Conversely, during mild cognitive impairment prior to more severe Alzheimer’s disease, the ability to perform vergence eye movement was weak (Jiménez et al., 2021). Perhaps vergence eye movements may be utilized in future studies as a classifier and predictor for mild cognitive impairment for Alzheimer’s disease (Hashemi et al., 2023). Parkinson’s disease also has a higher prevalence of convergence insufficiency (Lepore, 2006). This population also has greater delays in starting convergent and divergent eye movements (Hanuška et al., 2015). However, in specific brain insult, like strokes, only the parietal stroke locations are significantly related to vergence dysfunction (Anagnostou et al., 2021), further emphasizing the importance of the oculomotor aspect of the vergence system. Traumatic brain injuries, including patients who have suffered mild traumatic brain injuries such as concussions, have had a higher incidence of visual dysfunctions, especially vergence issues like convergence insufficiency (Alvarez et al., 2012; Suchoff et al., 1999). In fact, 50% of a sample of 160 people with traumatic brain injuries had convergence insufficiency, which was the most prevalent vergence disorder present, but there was also a prevalence of binocular instability, vergence excess, divergence insufficiency, basic exophoria, and basic esophoria, with some even having multiple convergence issues concurrently (Ciuffreda et al., 2007). Even years after injury, convergence insufficiency is found in 42% of the population with a history of brain injury (Cohen et al., 1989), while in the typical population, research reports 5–12% (Wajuihian and Hansraj, 2016; Rouse et al., 1999; Hussaindeen et al., 2017; Davis et al., 2016; García-muñoz et al., 2016; Letourneau and Ducic, 1988; Ovenseri-Ogbomo and Eguegu, 2016).

Children with neurological disorders like attention deficit hyperactivity disorder showed abnormal vergence eye movements (Bustos-Valenzuela et al., 2022). Perhaps vergence eye movement may serve as an objective indicator of cognitive decline or issues with attention. Degradation of vergence eye movements may in part be due to different processing of the incoming information (Solé Puig et al., 2015), making delineating the oculomotor vergence sensory and vergence oculomotor systems via fMRI a key next step to understanding the impact of attentional disorders for vergence eye movements.

### 4.5 Study limitations

The within-scan eye tracking is used to validate that the task is being completed by the participants. While binocular eye tracking is ideal for this experiment, prior fMRI studies on the vergence neural substrates use monocular eye tracking to confirm that the participants have performed the experimental task using monocular tracking (Fogt et al., 2023; Alvarez et al., 2021; Quinlan and Culham, 2007). Each of those functional imaging studies has also utilized binocular eye tracking outside of the MRI instrument. Similar to prior investigations, binocular eye tracking was performed outside the imaging experiment to confirm that the participants could perform binocular vergence eye movements and not disjunctive saccades. The training was conducted before the imaging experiment with the use of a proximal cue, the perception of depth shown in Figure 1D. A study limitation is monocular eye tracking during the imaging experiment. In addition, the EyeLink within the scanner has a spatial resolution of 0.25 degrees which does not permit a reliable assessment of microsaccades. However, with the monocular eye tracking, Figure 3 displays the right eye trace of a single participant over the course of the entire motor and sensory blocks. Figure 3 includes a velocity trace of the monocular eye position showing peak velocities less than 10 deg./s which is typical for vergence eye movements (Maxwell et al., 2010; Alvarez et al., 1998) and substantially slower than the peak velocities observed for saccadic eye movements (Guadron et al., 2022; Leigh and Zee, 2006). The significant spikes in the eye tracking trace are due to blinks, the frequent spikes could be due to eyelash noise as well.

### 4.6 Summary and future direction

The protocol described within this study has future clinical application for many patient populations to investigate the differences between the vergence neural substrates in a diseased or dysfunctional population compared to the results presented here for participants with normal binocular vision. In addition, this protocol information can be used to evaluate therapeutic interventions used to remediate clinical signs and symptoms in patients with vergence dysfunctions.

## Data Availability

The raw data supporting the conclusions of this article will be made available by the authors, without undue reservation.
